# Defining Skill in Manual and Manipulative Therapy: Perspectives from Physical Therapists

**DOI:** 10.3390/healthcare13172081

**Published:** 2025-08-22

**Authors:** William J. Hanney, Rachel A. Brown, Emily Lufsey, Georgia Newsome, Payne Sewnarine, Morey J. Kolber, Abigail W. Anderson

**Affiliations:** 1 Spine & Mobility Laboratory, School of Kinesiology and Rehabilitation Sciences, University of Central Florida, Orlando, FL 32816, USA; rachellola7@gmail.com (R.A.B.); emlufsey@gmail.com (E.L.); georgiaslay@gmail.com (G.N.); paynesewnarine@gmail.com (P.S.); abigail.wilson@ucf.edu (A.W.A.); 2 Department of Physical Therapy, Nova Southeastern University, Fort Lauderdale, FL 33328, USA; kolber@nova.edu

**Keywords:** manual therapy, physical therapy, skill, manual, manipulative therapy

## Abstract

**Background/Objectives**: A state-wide survey was conducted in Florida to evaluate skill in manual and manipulative therapy as a therapeutic intervention, explore its use in the field of physical therapy, and understand how physical therapists quantify skill to aid in creating a definition of what comprises manual and manipulative skills. A survey was developed utilizing the Delphi approach, which was entered into an electronic survey platform and distributed via email to physical therapists licensed in the state of Florida. Questions of the survey addressed the physical therapists’ demographics, education, training, use of MT, and Likert scales to quantify physical therapists’ perspectives on characteristics contributing to MT skills and how various characteristics contribute to skills in manual and manipulative therapies. **Results**: The response rate was 1.6% (307/19,523). Ninety-four percent of participants had more than 10 years of experience in physical therapy, and all respondents had at least 1 year of experience. Participants were 59.3% female and 40.7% male, with a mean age of 48 years (SD = 12.7). No association between gender and use of manual therapy was found (*p* = 0.44). Of respondents, 33.3% said they use manual and manipulative therapy on every patient, and 38.1% said they use it at least once a day. The most common specialty board certification that respondents held was in orthopedics. Among participants who believe that skill in manual and manipulative therapy is measurable, a Pearson’s correlation showed the following factors as key contributors of “skill” in manual therapy: therapeutic alliance, therapeutic touch, the ability to differentiate subtle nuances between grades I–IV, and adherence to evidence-based practice. **Conclusions**: The definition of skill found in this study could be used in future studies to determine how skilled MT interventions can influence patient outcomes. Future research should be conducted to discover how these aspects of manual and manipulative therapy can impact intervention results in patient care and how these definitions may influence physical therapy education.

## 1. Introduction

Manual and manipulative therapy is widely recognized as an effective intervention for managing musculoskeletal conditions, offering numerous benefits for patients [1,2,3,4,5]. Physical therapists use manual and manipulative therapy as an intervention for various musculoskeletal pathologies; however, outcomes associated with physical therapy treatment are variable based on a multitude of factors. These factors include therapists’ interaction with the patient, the patient’s confidence in the therapist‘s skill, the patient’s motivation to complete/participate in the intervention, and the patient’s expectations for treatment [4]. Another factor that may impact therapeutic outcomes is the patient’s confidence in the therapist’s skill. Patients value skills that enhance the patient–therapist interaction, including the ability to provide simple, clear explanations, motivation, encouragement to foster patient engagement, and adherence to treatment [6,7,8]. Understanding and quantifying the impact of manual and manipulative therapy on outcomes is crucial; however, there is a notable lack of evidence on the perspectives of current clinicians on the definition of “skill.” This survey study aims to explore physical therapists’ perceptions of skill in manual and manipulative therapy [8,9].

Facilitating discussion about the importance of skilled manual therapy is crucial to evaluating its role across various clinical settings. Evidence supports the effectiveness of spinal manipulation and mobilization in adults with acute, subacute, and chronic low back pain, as well as migraine, cervicogenic headache, and cervicogenic dizziness [10]. Additionally, manipulation and mobilization have been found effective for several extremity joint conditions [10,11,12,13], while thoracic techniques are beneficial for acute and subacute neck pain [10,14,15]. However, evidence for cervical manipulation or mobilization alone for neck pain of any duration remains inconclusive [10]. Similarly, there is limited or inconclusive evidence for the use of manipulation or mobilization in conditions such as mid back pain, sciatica, tension-type headache, coccydynia, temporomandibular joint disorders, fibromyalgia, premenstrual syndrome, and pneumonia in older adults [10].

The value of manual therapy can be significantly influenced by a patient’s confidence in the physical therapist’s skill [16,17,18]. Factors, such as therapist communication, professionalism, and demeanor, influence patient trust. For instance, clinicians’ attire, whether a white coat or casual attire, has been shown to affect patient confidence [19]. In patients undergoing total shoulder arthroplasty, confidence in the treatment outcomes is often shaped by their expectation of recovery prior to surgery [20]. Similarly, a therapist’s enthusiasm and optimism can enhance patient confidence [21]. Patient satisfaction is also influenced by characteristics of the physical therapist, such as professionalism, a friendly attitude, and effective communication [6]. Interestingly, a study showed that patients with low back pain favored clinicians with a reputation for their technical skills over those known for interpersonal skills [18,22,23], further emphasizing the multifaceted nature of patients’ perceptions.

It is important to recognize that patients’ values and preferences, while important, do not inherently validate a treatment’s effectiveness [10,24]. A patient may express satisfaction with a treatment even if it lacks clinical efficacy [10,25]. Manual and manipulative therapy research provides valuable evidence of its effectiveness through systematic observation and efforts to minimize bias, ensuring that findings can be reliably applied to appropriate patient populations [10].

Therefore, the importance of skilled manual and manipulative therapy should be studied to understand the impact it has on the outcomes. Skilled manual and manipulative therapy is a valuable component of physical therapy, yet its definition remains ambiguous, complicating efforts to quantify its effectiveness. This study aims to evaluate physical therapists’ perceptions of skilled manual and manipulative therapy, identify its utilization across clinical settings, and explore factors contributing to skill. The result of this study will aid in identifying the definition of skill in manual and manipulative therapy in the field of physical therapy and what factors physical therapists believe contribute to skill.

## 2. Materials and Methods

### 2.1. Study Development

A two-round modified Delphi consensus process was employed to identify key questions for evaluating the role of skill in manual therapy. In the first round, initial questions were generated through consultation with two certified manual therapists and one non-certified manual therapist. These questions were reviewed for thematic consistency, and those with overlapping content across all three reviewers were retained. The refined set of questions was then presented to a broader group of clinicians, who rated each item based on perceived importance. The 12 highest-ranked questions were selected for inclusion based on these ratings [26,27] (Appendix A).

Given that the primary aim of this study was to examine clinicians’ perceptions of skill in manual therapy, we employed a Likert scale to operationalize this inherently subjective construct. While perceptions of skill are qualitative in nature, the use of a Likert scale allowed for the systematic collection of ordinal data, enabling quantifiable analysis of subjective perspectives. This methodological approach was selected to enhance the interpretability and consistency of responses while preserving the nuance of individual perception. We acknowledge the limitations inherent in attempting to quantify a subjective phenomenon, and this is addressed further in the discussion of study limitations.

The electronic survey was created in Qualtrics, which allowed for electronic distribution of the survey via a URL link. Data was collected and transferred to JASP (version 0.19.3) for analysis. The final survey was adjusted to take only three minutes for an increased incentive for completion. The survey contained 12 questions and was available in English. The institutional review board at the University of Central Florida approved the study design and questionnaire.

### 2.2. Study Subjects

The electronic survey was sent to all licensed physical therapists (PTs) in the state of Florida. Emails of licensed PTs were obtained through the Florida Department of Health, accessed via the Florida State Health Care Practitioner Data Portal. Potential subjects were excluded from participating in the survey if their license was pending, expired, or if they did not attach a valid email address. Participants who qualified for this study were sent an email with an invitation and a flyer to participate in the survey through an electronic link. The email was sent by a valid email address from an academic institution. “Test” emails were sent to the researchers to ensure the survey email would not be sent to spam or junk folders. Four rounds of about 5000 emails were sent until all 19,523 emails were sent over 6 weeks. Emails were divided among four researchers and sent to 99 recipients per email. Recipients received a reminder email to complete the survey one week after the initial email was sent. The reminder email was sent from the same address as the original email with the survey link and flyer.

### 2.3. Study Design

The participants included licensed physical therapists practicing in the state of Florida. The Florida Department of Health reported 19,523 physical therapists who had an active status on their license, a valid email address, and were able to read English. An electronic survey was sent to all 19,523 licensed physical therapists in the state of Florida who were 18 years or older and spoke English. The survey was developed based on the available literature reviews completed by researchers and consultations with physical therapy experts from the academic and professional fields. The final 19-item questionnaire required less than three minutes to complete. The survey was divided into three sections. Section I addressed demographics and use of manual and manipulative therapy (nine items); Section II addressed experience (two items); Section III addressed defining skill i(eight items). The characteristics of skill were assessed using a 10-point Likert scale ranging from 1 (strongly disagree) to 10 (strongly agree). The Qualtrics survey was created, trialed, and reviewed by content experts.

### 2.4. Data Analysis

A power analysis conducted on G-Power indicated the required sample size to achieve 80% power for detecting a medium effect, at a significance criterion of α = 0.05, was N = 305 for the ANOVA test. Survey responses were excluded if the respondent did not complete Section III of the survey. Statistical analysis was completed using JASP software version 0.18.2.0. The statistical analyses included Pearson correlation, Linear regression analysis, and Chi-squared test, with alpha set at 0.05.

## 3. Results

The total number of respondents who fully completed the survey was 307. Participants were 59.3% female and 40.7% male (Table 1), with a mean age of 48 years (SD = 12.1). Race and ethnicity of respondents were largely white (74.3%); however, African American (3.6%), American Indian/Alaskan native (4.6%), Asian (9.4%), and Hispanic/Latino (3.5%) were also included (Table 2). The average number of years in clinical practice among respondents was 22, with the minimum being one year and the maximum 50 years (M = 22, SD = 12.4). Participants without a board certification included 74.6% (229/307) of participating clinicians, and participants with a board certification included 25.4% (78/307) of responding clinicians (Table 3). Entry-level degree type among respondents included BSPT 34.5% (106/307), MPT 13% (40/307), MSPT 15.6% (48/307), and DPT 36.8% (113/307) (Table 4). Of the respondents, only 9.4% (29/307) held a fellowship in the American Academy of Orthopedic Manual Physical Therapy.

When asked how frequently the physical therapist uses manual therapy, 31.3% of respondents reported using manual and manipulative therapy on every patient, and 38.1% answered, “at least once a day” (Table 5). Through completion of a Chi-squared analysis, no association was found between gender and use of manual therapy (*p =* 0.44). It was also found that there is an association between having a specialization in orthopedics (*p* = 0.019) or sports (*p* = 0.014) and increased use of manual and manipulative therapy. Conversely, there is an association between having a specialization in cardiopulmonary and neurology and not using manual and manipulative therapy *(p* = 0.006 and 0.006). Additionally, there is an association between having a tDPT and frequent use of manual therapy (on every patient and at least once a day) (*p* = 0.028) (Table 6).

Therapists who believe skill is measurable in manual and manipulative therapy included 75.3% of respondents (Table 7). Pearson correlation revealed respondents who believe skill is measurable also believe therapeutic alliance, therapeutic touch, differentiating subtle nuances, and evidence-based practice are the factors that highly contribute to skill in manual and manipulative therapy (Table 8). Linear regression analyses revealed that years of clinical practice are positively correlated with the belief that confidence contributes to skill in manual and manipulative therapy (*p* < 0.001). Additionally, an ANOVA and linear regression revealed that years of clinical experience are positively correlated with use of manual and manipulative therapy (*p* = 0.018, R^2^ = 0.044) (Table 9), suggesting that with an increase in years of clinical experience, there is increased use of manual and manipulative therapy (Table 10).

## 4. Discussion

Manual and manipulative therapy is a commonly used intervention for various musculoskeletal injuries. It is important to consider how treatment interventions like manual and manipulative therapies will affect patient outcomes. There appears to be a paucity of evidence that defines “skill” in manual therapy, especially when comparing novice and experienced clinicians. It is suggested that post-graduate training, such as residency programs and continuing education courses, is one method of enhancing one’s skills in a physical therapy specialty. Manual and manipulative therapy, a crucial specialty tool used in physical therapy, should be carefully and purposefully applied in a skillful manner. Unskilled manual therapy may not yield the desired effect, worsening and/or inaccurately distorting patient outcomes. With the goal of improving patient outcomes in mind, we aimed to help define “skill” in manual therapy from the perspective of physical therapists through a survey.

According to the perspectives of licensed physical therapists in Florida, skill is affected by therapeutic alliance and therapeutic touch. Arrigoni et. al state that manual therapists who can manage patients’ beliefs are able to achieve positive feedback, promoting homeostatic abilities and reducing allostatic overload [28]. In Arrigoni’s study, clinicians were surveyed with the intent to establish meaningful key points of therapeutic alliance in musculoskeletal care. Three primary categories were reported as significant by clinicians: creating a meaningful dialogue—active listening and understanding expectations, promoting active patient participation—goal sharing, person-centered care, and synchronization—therapeutic touch and patient’s feedback [23,29]. Clinicians revealed that the therapeutic touch was a key aspect in building therapeutic alliance, and it encourages a sense of care and understanding, highlighting the importance of a patient-centered approach [28]. Manual therapy is a specialty tool that includes frequent, purposeful, and skillful “touch”. Ensuring therapeutic touch and alliance are prioritized may improve outcomes more than solely relying on one’s own experience and perspectives, as these can be considered factors that affect skill.

This study also found statistical significance in differentiating subtle nuances between different grades of joint mobilization, which contributes to “skill” in manual therapy. Although various schools of thought differ in manual therapy when applying joint mobilizations, it has been shown that different grades I–IV are distinguishable through different applications of force [30,31,32]. According to Petersen et al., providing objective feedback to students may be a way to achieve more consistent and accurate force application during manual therapy maneuvers [33]. This suggests that using joint force measurement devices could assist in enhancing joint mobilization skills beyond solely relying on expert clinician demonstration and monitored breakout sessions. If emphasis is placed on accurately learning and differentiating grades of joint mobilizations, manual therapy expertise can objectively be attained.

By establishing a definition for “skill” in manual therapy, this study has laid the foundation for an objective outcome measure to be created with metrics that will be sensitive/specific for detecting “skillful” manual therapy. All settings in physical therapy have experts who carefully and competently apply “skill” to provide their patients with the best possible outcome. Manual therapy is no less significant when measuring skill. We must aim to be meaningful through intent, dosage, application, patient interaction, tissue healing times, and technique. Britnell et al. describe three key points that demonstrate the necessity for specialty practice: (1) procedures have become more complex and effective, requiring more specialty training; (2) specialization produces better outcomes, and (3) patients will drive further specialization as health literacy of improved outcomes with specialty practice increases [34]. As disease etiology evolves, the need for specialists to meet patients’ physical and medical complexities with “skillful” manual therapy increases, highlighting the importance of defining skill in manual therapy. Future research may consider the development of a standardized tool to assess perceived clinician skill in manual therapy. Such a tool could facilitate more consistent measurement across studies and support efforts to better understand the relationship between practitioner skill and treatment outcomes in manual therapy interventions.

There are a few limitations to acknowledge. Although we used a Likert scale to quantify clinicians’ perceptions of skill, this approach attempts to objectify a fundamentally subjective construct. As such, responses may be influenced by individual interpretation, limiting the precision of measurement. While the respondents were required to have an active license in Florida, their education could have been from any PT program across the world, with variations in manual therapy curriculum and teaching faculty credentials. The results of this study cannot be generalized to practitioners outside of Florida or the U.S. Also, Formal training and degree requirements vary in different countries; therefore, results reporting educational level may not be relevant to a practitioner outside of the U.S. Another limitation includes how survey questions were asked. Due to the categorical nature of survey studies, there were limitations in how data could be analyzed in JASP. Finally, the low response rate (1.6%) was a limitation of this study; however, adequate power was achieved based on a priori power analysis.

## 5. Conclusions

This study aimed to assess the current opinions and perspectives of physical therapists on various aspects of manual and manipulative therapy practice and whether those aspects may be affected by skill. A survey study was used to gain insight into the viewpoints of physical therapists to construct a definition of skill in manual therapy.

This study advances the field of physical therapy by evaluating perceived qualities that influence utilization and skill of manual and manipulative therapy in clinical practice. Through the collection and analysis of survey data, it can be concluded that differences exist in use of manual and manipulative therapy, and skill in manual therapy can be defined as, effectively establishing a therapeutic alliance, using proper therapeutic touch during a technique, the ability to differentiate subtle nuances between grades I–IV, and adherence to evidence-based practice.

## Figures and Tables

**Table 1:** Sex of Survey Respondents.

Biological Sex	Number of Respondents	Percent (%)
Female	182	59.3
Male	125	40.7

**Table 2:** Demographics of Survey Respondents.

Race and Ethnicity	Number of Respondents	Percent (%)
African American or Black	11	3.6
American Indian or Alaskan Native	14	4.6
Asian	29	9.4
Hispanic/Latino	11	3.5
White (Not Hispanic)	228	74.3
Other	14	4.6

**Table 3:** Frequencies for “Do you have a Board Certification?”.

	Number for Respondents	Percent (%)
Yes	78	25.4
No	229	74.6

**Table 4:** Frequencies for Entry-Level Degree Type.

	Number of Respondents	Percent (%)
BSPT	106	34.5
DPT	113	36.8
MPT	40	13.0
MSPT	48	15.6

**Table 5:** Frequency of Use of Manual Therapy.

How Frequently Do You Use Manual Therapy in Practice?	Frequency	Percent (%)
I do not use manual therapy in practice	25	8.1
At least once a day	117	38.1
At least once a week	27	10.1
At least once a month	31	8.8
At least every 6 months	11	3.6
On every patient	96	31.3

**Table 6:** Estimated Chi-Square Test Results for Associations with Use of Manual and Manipulative Therapy.

Comparison	Chi-Square Association	*p*-Value
Gender vs. Use of Manual Therapy	No	0.44
Orthopedics vs. Use of Manual/Manipulative Therapy	Yes	0.019
Sports vs. Use of Manual/Manipulative Therapy	Yes	0.014
Cardiopulmonary vs. Use of Manual/Manipulative Therapy	Yes (Negative association)	0.006
Neurology vs. Use of Manual/Manipulative Therapy	Yes (Negative association)	0.006
tDPT vs. Frequent Use of Manual Therapy (daily/every patient)	Yes	0.028

**Table 7:** The Perspectives on Whether “Skill” in Manual Therapy is Measurable.

Item, Item Response	Yes (%)	No (%)
“Do you feel that ‘Skill’” is measurable?	230 (75)	77 (25)

**Table 8:** Estimated Pearson Correlations Between Belief That Skill Is Measurable and Key Contributing Factors in Manual and Manipulative Therapy.

Variable	Skill Is Measurable
Belief in Therapeutic Alliance as a Contributor	r = 0.62
Belief in Therapeutic Touch as a Contributor	r = 0.58
Belief in Differentiating Subtle Nuances	r = 0.66
Belief in Evidence-Based Practice as a Contributor	r = 0.71

**Table 9:** Estimated Linear Regression Results: Years of Clinical Experience Predicting Beliefs and Use of Manual and Manipulative Therapy.

Outcome Variable	Predictor	β (95% CI)	*p*-Value	R^2^
Belief that Confidence Contributes to Skill	Years of Clinical Practice	0.42 (0.30–0.54)	<0.001	0.18
Use of Manual and Manipulative Therapy	Years of Clinical Practice	0.21 (0.04–0.38)	0.018	0.044

**Table 10:** The Four Factors that Contribute to Skill in Manual Therapy.

Therapeutic Alliance
Therapeutic Touch
Differentiating Subtle Nuances
Evidence-Based Practice

## Data Availability

The original contributions presented in this study are included in the article. Further inquiries can be directed to the corresponding author.

## Appendix A

Survey questions:

Section 1: Demographics

1. What is your age (years)?
2. What is your biological sex?
  - Male
  - Female

3. What is your race/ethnicity?
  - African American or Black (Not Hispanic)
  - American Indian or Alaskan Native
  - Asian
  - Hispanic/Latino
  - Pacific islander or Native Hawaiian
  - White (Not Hispanic)
  - Other

4. For each of the following, write down the number of years of experience you have in each field.
  - Academic Institution (Postsecondary)
  - Acute Care Hospital
  - Health and Wellness facility
  - Hospital-Based Outpatient Facility or Clinic
  - Home Care/Patient’s Home
  - Industry
  - Inpatient Rehab Facility
  - PT in skilled nursing
  - Private Outpatient Office or Group Practice
  - Research Center
  - School System (Preschool/Primary/Secondary)
  - Skilled Nursing Facility (SNF)/Long Term Care
  - Travel PT

5. How many years of clinical practice in total do you have?
  - ____

6. What is your entry-level physical therapy degree?
  - BSPT
  - MSPT
  - MPT
  - DPT
  - Do you have a post-professional degree?
    - tDPT
    - PhD
    - EDD
    - Other

7. Do you have a board certification?
  - Yes
    - Which specialty area is it in?
      1. Cardiovascular and pulmonary
      2. Clinical electrophysiology
      3. Geriatrics
      4. Neurology
      5. Oncology
      6. Orthopedics
      7. Pediatrics
      8. Sports
      9. Women’s health
      10. Wound management
  - No

8. Do you hold a fellowship in the American Academy of Orthopedic Manual Physical Therapy?
  - Yes
  - No

9. How frequently do you use manual therapy in practice?
     “I use manual therapy…”
  - on every patient
  - at least once a day
  - at least once a week
  - at least once a month
  - at least once every 6 months
  - I do not use manual therapy in practice

Section 2: Manual therapy Experience

10. Please select which of the following manual therapy organizations you are certified in.
  - Maitland-Australian Physiotherapy Seminars
  - The McKenzie Method of Mechanical Diagnosis and Therapy
  - The Mulligan Manual Concept
  - The Paris Approach
  - The Kaltenborn-Evjenth Othopedic Manipulative Therapy Concept
  - None
  - Other (please specify):

Section 3: Defining skill in manual therapy

11. Do you feel that skill is measurable?
12. Please rate each statement below by how much you think it contributes to skill in MT.
  - 0-----1-----2-----3-----4-----5-----6-----7-----8-----9-----10
  - Establishing a therapeutic alliance between patient and clinician
  - Clinician confidence in the MT technique being applied
  - Therapeutic touch applied during the MT technique
  - Differentiating subtle nuances in manual therapy grades (Grades I-V)
  - Using evidence-based practice to determine which patient presentations would benefit from manual therapy techniques.
  - Assessing patient reactivity to manual therapy and choosing to continue or discontinue MT use in POC
  - Advanced clinical reasoning
